# Supplementary material for: A Novel p-Type ZnCoxOy Thin Film Grown by Atomic Layer Deposition
Source: Nanomaterials (Basel). 2022 Sep 27;12(19):3381. doi: 10.3390/nano12193381 (PMC9565241; doi:10.3390/nano12193381)
Supplement: Supplementary file 1 [file nanomaterials-12-03381-s001.zip › nanomaterials-1882777-supplementary.pdf]

Supplementary Materials

A Novel p-type $ZnCo_xO_y$ Thin Film Grown by Atomic Layer Deposition

Leyi Li [1], Zhixin Wan [1,*], Quan Wen [2], Zesheng Lv [2] and Bin Xi [1,*]

[1] School of Materials Science and Engineering, Guangzhou Key Laboratory of Flexible Electronic Materials and Wearable Devices, PFCM Lab, Sun Yat-sen University, Guangzhou 510006, China
[2] School of Electronics and Information Technology, Sun Yat-sen University, Guangzhou 510006, China
* Correspondence: wanzhx@mail.sysu.edu.cn (Z.W.); xibin3@mail.sysu.edu.cn (B.X.)

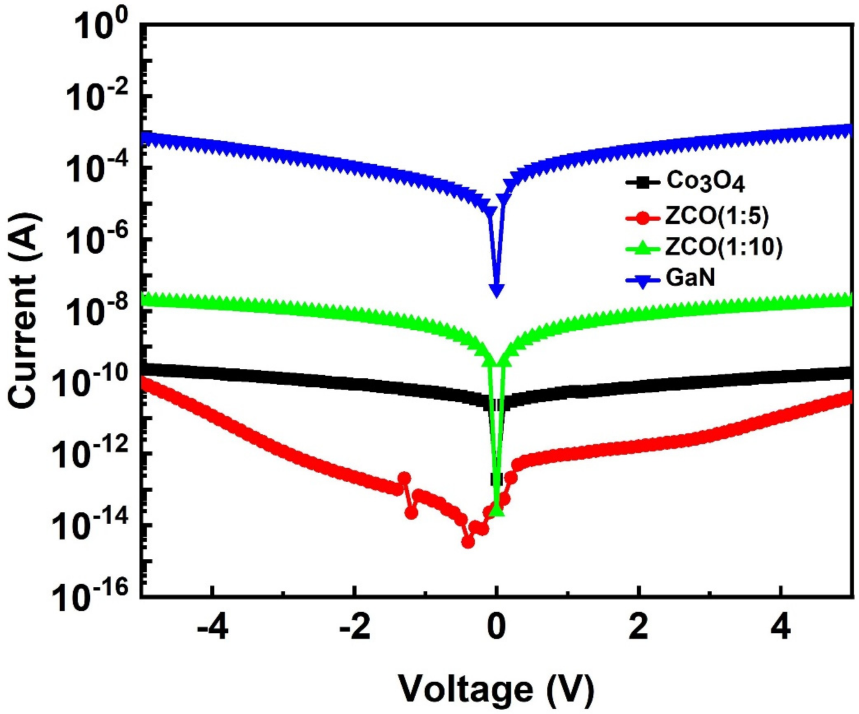

Figure S1. Ohmic contact of the p-n diodes fabricated by pure Co_3O_4 film, GaN film and $ZnCo_xO_y$ films with different deposition ratio.

Table S1. The composition of ZCO (1:10) film obtained from XPS analysis.

Samples	Zn (at. %)	Co (at. %)	O (at. %)	C (at. %)
ZCO(1:10)	18.5	30.5	44.4	6.6

Table S2. The electrical properties and bandgap of pure ZnO, pure Co_3O_4 and $ZnCo_xO_y$ films with different deposition ratio.

Samples	Carrier concentration (cm^{-3})	Mobility ($cm^2V^{-1}s^{-1}$)	Resistivity (Ω·cm)	E_g (eV)
ZnO	5×10^{18}	1.03	1.18	3.25
Co_3O_4	3.49×10^{18}	1.76	1.02	1.85
ZCO(1:1)	3.05×10^{18}	8.91	0.23	3.2
ZCO(1:5)	3.09×10^{17}	16.59	1.22	--
ZCO(1:10)	2.46×10^{19}	1.09	0.23	2.15

Table S3. The ratio of forward-to-reverse current (I_f/I_r), forward turn-on voltage and ideality factor of the p-n junctions prepared by pure Co_3O_4 and $ZnCo_xO_y$ (1:5, 1:10) thin films.

Sample	I_f/I_r(±5V)	V_{ON}(V)	η
Co_3O_4	6.53×10^6	0.5	1.4
ZCO(1:5)	1.75×10^7	1	1.3
ZCO(1:10)	1.94×10^9	2.4	1.5
